# Continuing professional development on climate change and primary care in Africa: Qualitative study

**DOI:** 10.4102/phcfm.v17i1.4916

**Published:** 2025-09-29

**Authors:** Robert Mash, Christian Lueme Lokotola

**Affiliations:** 1 Division of Family Medicine and Primary Care, Faculty of Medicine and Health Sciences, Stellenbosch University, Cape Town, South Africa

**Keywords:** climate change, planetary health, continuing professional development, learning needs, primary care

## Abstract

**Background:**

Climate change is impacting health and healthcare in Africa. Primary health care can improve community resilience, but only if the workforce is prepared. Pre-service training does not yet address climate change, so continuing professional development (CPD) is needed.

**Aim:**

This study aimed to evaluate what primary care providers in sub-Saharan Africa need to know about building climate-resilient facilities and services, and how their learning needs should be addressed.

**Setting:**

The Primary Care and Family Medicine (PRIMAFAMED) network in sub-Saharan Africa.

**Methods:**

A descriptive exploratory qualitative study purposefully selected members of the network who had published on their experience of climate change. Snowball sampling was used to identify additional informants. Data were analysed with ATLAS.ti and the framework method.

**Results:**

Nine respondents from eight countries across Africa identified six major learning needs: (1) awareness of the pathways that link climate change to health and social effects and changes in the management of diseases, (2) management of diseases linked to exposure to extreme heat, (3) development of a community-orientated primary care approach that includes attention to environmental determinants of health, (4) disaster preparedness and management, (5) how to make your facility and services more climate resilient and (6) how to educate patients and communities on climate related health issues. Most respondents supported web-based approaches to CPD in their contexts.

**Conclusion:**

Key learning needs were identified and will be further quantified and validated in a cross-sectional survey.

**Contribution:**

The findings will inform the development of CPD on planetary health for primary care providers in sub-Saharan Africa.

## Introduction

Climate change is one of several interconnected ecological crises affecting our planet and intersects with health via three broad pathways.^1^ Firstly, climate change may have direct health and social effects through changes in food production, water quality and quantity, exposure to infectious diseases and extreme weather events. Secondly, climate change can disrupt health facilities and services, further increasing the vulnerability of communities. Thirdly, health facilities and services may be responsible for greenhouse gas emissions that exacerbate climate change.

Primary health care (PHC) can mediate the impact of climate change on health.^2^ It is the portal of entry to the health system and can improve the preparedness and resilience of communities.^3^ It is designed as the cornerstone of health systems, providing comprehensive care and contributing to universal health coverage.^4^ It goes beyond primary care services, to also empower communities and support multisectoral action on the determinants of health and well-being.^5^

Primary health care, therefore, has the potential to increase the resilience of communities,^3^ but to do this PHC must be resilient and of high quality.^6^ The primary care workforce is a key component of building this preparedness and resilience, and yet many primary care providers have no specific training on climate change. A recent scoping review on PHC and climate change in Africa identified the learning needs of the PHC workforce as a key research question and knowledge gap.^7^

The PRIMAFAMED network (an institutional network of departments of family medicine and primary care in sub-Saharan Africa)^8^ held a workshop on climate-related educational needs. The workshop identified the need to investigate the continuing professional development (CPD) needs of primary care providers and to determine the best educational approaches. Most of the frameworks and guidelines on planetary health education focus on formal curricula and not CPD.^9,10^ The aim was to evaluate what members of the PRIMAFAMED network in sub-Saharan Africa perceive as the learning needs of primary care providers with regard to building climate-resilient facilities and services, and how their learning needs should be addressed.

## Methods

### Study design

This was a descriptive exploratory qualitative study. This study will form the first part of an exploratory sequential mixed methods study.

### Study setting

Public sector primary care providers in Africa are usually community health workers, nurses or clinical officers (physician assistants) and are supported by family doctors. This study made use of the PRIMAFAMED network and its affiliated journal, the *African Journal of Primary Health Care and Family Medicine* (PHCFM), to identify key informants. The PRIMAFAMED network includes departments of family medicine and primary care from 25 countries and 40 institutions in sub-Saharan Africa.^8^ These departments are involved in training family doctors as well as nurses and clinical officers.

### Study population

Key informants were defined as members of PRIMAFAMED who had directly experienced the impact of climate change on PHC in sub-Saharan Africa.

### Sample size and sampling

Purposeful sampling was used, with the intention of maximum variation in the informants’ experience of climate hazards (cyclones, flooding, extreme heat and drought) and their proximal effects on food production, water and infectious diseases. Key informants were identified from the authors of a special collection on the effects of climate change on PHC in Africa that was published in the PHCFM. We then selected one author from each of the following countries: South Africa, Malawi, Nigeria, Kenya, Zimbabwe and Mozambique. This gave an initial sample size of six key informants. Additional informants were identified through snowball sampling. Saturation was implied by no new data emerging from the last two interviews.

### Data collection

Semi-structured interviews were supported by an interview guide. The opening question asked how climate change impacted PHC facilities and services and what new knowledge and skills were needed. Specific topics could be explored further:

- Understanding the ecological crises and the health and social effects
- Changes to health programmes and services as a result of climate change
- Emergency preparedness and disaster management
- Vulnerability and risks of the community served
- Advocacy in the health system and broader society
- Implications for facility’s infrastructure and functioning
- Local approaches to CPD

Interviews were virtual, recorded in English, conducted by one of the authors, and lasted 30 min – 60 min.

### Data analysis

Verbatim transcripts were checked against the recordings. The analysis was conducted by both authors, with the help of ATLAS.ti version 23, using the framework method:^11^

- *Familiarisation*: reading the transcripts and listening to the tapes to identify key issues that could be coded.
- *Coding index:* codes were defined and organised into categories.
- *Coding:* the researchers applied the codes across all the transcripts.
- *Charting:* code families were created, based on the categories, and a report was created.
- *Interpretation*: the reports were interpreted to identify key themes and any relationships between themes.

### Trustworthiness

Christian Lueme Lokotola was a researcher in planetary health, but not experienced in qualitative methods. He was supervised by RM, who was a family physician and experienced qualitative researcher. Both researchers, had no clearly pre-defined ideas on the learning needs of primary care providers. Neither researcher had organisational links or close relationships to the key informants. Both researchers were aware of the need to be conscious of their own beliefs and how these might influence the interviews and analysis.

### Ethical considerations

Ethical clearance to conduct this study was obtained from the Stellenbosch University Health Research Ethics Committee (No. N24/01/003).

## Results

The characteristics of the nine respondents are shown in Table 1. Six broad themes are presented below and supporting quotations from the data are given in Table 2. Key informants had not conceptualised the CPD needs of primary care providers per se and mostly spoke about the effects of climate change on PHC. The CPD needs could then be extrapolated from these experiences and observations. The following themes were identified:

- Understanding climate change and the health system
- Issues with diseases and conditions
- Issues with emergency preparedness and disaster management
- Issues with routine service delivery
- Issues with climate resilience
- Approaches to education

**TABLE 1:** Participants’ characteristics.

Participant	Age	Gender	Role/Job	Country
P1	50	Female	Family physician	South Africa
P2	68	Female	Public health practitioner	Botswana
P3	34	Male	Medical practitioner	Malawi
P4	34	Male	Public health practitioner	Zimbabwe
P5	36	Female	Family physician	Kenya1
P6	62	Male	Public health practitioner	Kenya2
P7	37	Female	Medical practitioner	Mozambique
P8	53	Male	Family physician	Nigeria
P9	55	Male	Family physician	Ghana

**TABLE 2:** Selected quotations that support the themes.

Theme	Selected quotations from the data
Understanding climate change and the health system	‘So I think first and foremost, understanding the implications of climate change and health, as I mentioned that sometimes we do like, we raise awareness among health professionals and you know interactions with them actually realizing that a lot of people are not, they do not consider climate change to be a conversation that’s really needed to be having in the health space.’ (Zimbabwe)
Issues with diseases and conditions	‘We’re also seeing a change in conditions related to asthma and other allergic diseases presenting in a primary health care setting.’ (Malawi) ‘This [*heat*] resulted in an increase of babies presenting with heat rashes, patients presenting with dehydration.’ (South Africa) ‘The major, the most common diagnosis was with gastroenteritis.’ (Malawi) ‘That specific species of rodent increased in numbers, moving into houses because of flooding. Then you have contact with humans and then you have Lassa fever.’ (Nigeria) ‘The duration of snake-human encounters increased over the period, because the snakes had enough time to forage for food and breed, because of the hot weather.’ (Ghana) ‘They are diseases that we thought “no it was forgotten,” in the emergence disease they are coming even with common diseases like respiratory disease… it was polio, pellagra, upper respiratory disease.’ (Mozambique)
Issues with emergency preparedness and disaster management	‘We did not have disaster preparedness plan. It was more of like a reaction. We went on the ground after it had already happened. So, we have a government system that provides disaster management, but we do not prepare.’ (Malawi) ‘One has been the flooding and tornado that happened 2 weeks ago, so during these events where the clinics are inundated with patients coming in with physical injuries from the weather.’ (South Africa) ‘Primary healthcare providers need to be equipped with emergency skills like managing cardiac arrest, managing trauma, how to organize a mass casualty response, which is a skill of its own, and it is something that needs to be practiced and guidelines set within each facility.’ (Kenya1) ‘We need to go home by home or that affected areas and inform please that things will happen in this area, and you be prepared in this in this way and you need to move to a secure place that is located at this point.’ (Mozambique) ‘Important things were the drugs were not enough. Actually, when we went there, we had to get support from other providers like Partners in Health because even the government was almost like it was choked. It didn’t manage to cope.’ (Malawi) ‘Because there are some areas where infrastructures were destroyed because of the cyclone, so people had to move to find clinics.’ (Malawi) ‘So gender based violence went up because people are staying in camps. So mothers and daughters and fathers not related were staying in just the school block.’ (Malawi) ‘I think we underestimate the impact of emotional and psychological trauma to communities and patients when they’re events such as this.’ (South Africa)
Issues with routine service delivery	‘When we have this disruption of services, this can result in nonadherence to medication with patients with hypertension, diabetes, HIV and that can affect their health and result in complications.’ (South Africa) ‘Primary healthcare provider should not feel that his job starts and ends sitting in his room in the facility; that he should understand that his job is more global…So imagine that the doctor, the clinical officer, the nurse, actually people know that he is in the dispensary, but he actually spends time in the community, having community meetings, talking to them in the villages. That would definitely have a greater impact.’ (Kenya2) ‘They also should have competencies in doing community awareness. Yeah. Empowering the community in terms of ways in which to adapt to the effects of climate change on their health, they should have competencies in terms of constant screening of community, or the needs within the community, cause sometimes it keeps changing with the changing climate or the changing weather.’ (Kenya1) ‘And what the residents of that place would say is that accessing healthcare services in the afternoon was a challenge. Yeah, because having to check to a facility that is about 10–15 kilometers away from their home state in the scorching sun was difficult. So most people would tend to go to the health facilities in the morning and in the afternoon then they would opt to just stay in their houses, stay indoors.’ (Kenya1)
Issues with climate resilience	‘Sitting in a queue outside the clinic [*in the heat*] and the queue isn’t in the shade, then just sitting in that queue will put people at risk.’ (Botswana) ‘But we discussed with a lot of people around it, they don’t think about it in the in the context of climate change. The only think about it in the context of solving a power issue.’ (Zimbabwe) ‘The dispensary, the water source is water trucking, so they request from town, from Malindi or other places and then it comes to this place. So they cannot come. Now the place is without any clean water. The dispensary running on river water, which is contaminated and dirty.’ (Kenya2) ‘And you know, doctors and nurses, you say to them the temperatures are going to go up and they say we’ll turn on the air conditioning now in Zimbabwe most places don’t have air conditioning, and they’re now beginning to put them in because there’s no public campaign to say air conditioning makes this worse.’ (Botswana)
Approaches to education	‘Maybe even integrating climate change and its impact on health within the curriculum for undergraduate students, so that when they do graduate and eventually, you know, a medical officer is basically a primary care provider. So integrating education on climate change and its effects on health and practical examples within the curriculum for undergraduate students can really help with advancing the knowledge on this topic and awareness.’ (Kenya1) ‘OK, so I believe that they should have competencies in managing respiratory tract illnesses. Number one, they should have competencies in identifying and emerging and managing water borne illnesses. This here I refer to faecal oral transmitted infections. So, diarrhea due to any reasons, those competencies are highly important.’ (Kenya1) ‘Yes, generally right now there are lots of like these online courses that are available. People over there prefer that if there is in person training, but a big incentive for that is they’re getting per diems for that, and that’s spoils it all.’ (Kenya2) ‘People would you know, they wouldn’t have a laptop or something. It would be difficult for them to be like typing assignments, but they, you know, they can engage online on their cell phone, they could be listening to things they could download and read, things they could engage in a discussion and that that would be easier for them.’ (Kenya2) ‘Journals, journals. Gonna be very good resources because you can go attending a journal as long as it’s a primary health care provider. As journal you go and find materials that are related to climate change.’ (Malawi) ‘Let me be clear about the culture of my nation, I would say the Ghanaian is a person of certificates.’ (Ghana)

HIV, human immunodeficiency virus.

### Understanding climate change and the health system

Respondents were aware of climate change and other aspects of the ecological crisis such as deforestation and pollution (e.g. heavy metals, plastic). Most respondents reported that primary care providers lacked understanding of how climate change impacts health, healthcare facilities and services. In a few countries that had experienced extreme weather impacts, awareness of these ecological issues was emerging.

Respondents commented that primary care providers needed a supportive policy environment to prepare for the shocks and stressors of climate change. Primary care providers could advocate for appropriate policy development and guidelines, although this was not yet happening, and they might need training in appropriate skills. Health systems should look at monitoring environmental indicators, climate sensitive diseases and creating both surveillance and early warning systems.

### Issues with diseases and conditions

Respondents spoke of a range of health effects related to heat, drought and reduced water quality and quantity. Heat stroke, exhaustion and related dehydration were not that common as people had adapted their behaviour. In one country there was an association with increased snake activity and envenomation. Desertification could also be associated with worsening dust storms and acute respiratory problems. The failure of crops, drying up of lakes and dams and poor water supply were associated with malnutrition and diarrhoea. Respondents also mentioned effects on allergies (e.g. asthma), infectious diseases (e.g. malaria) and chronic conditions such as diabetes (e.g. foot ulcers and skin problems). Certain groups in the population were identified as more vulnerable, including the elderly, children, those with disabilities and pregnant women.

During acute events such as cyclones and floods, respondents identified gastroenteritis as a key problem, including the emergence of cholera due to poor sanitation. In one country flooding caused rats to enter houses and create Lassa fever outbreaks. Access of people with chronic conditions to medication could also be disrupted with implications for control of diseases such as human immunodeficiency virus (HIV), diabetes and hypertension. In these dire circumstances old diseases could remerge such as polio and pellagra.

These events were associated with many psychosocial and mental issues, including gender-based violence. Mental health issues, such as depression and anxiety, were seen as common after losing family members, property and livelihoods.

### Issues with emergency preparedness and disaster management

Most respondents reported that PHC facilities did not have adequate emergency preparedness and disaster management plans. They reacted and responded in a pragmatic manner when the event occurred. Some knew of disaster plans but did not apply them when the disaster happened. In some places primary care was not considered when making disaster plans that were more orientated towards road traffic accidents or infectious disease outbreaks. Health services needed to spend time planning and preparing how they will respond to climate events.

Respondents reported peaks of healthcare demands during climate change events. Facilities lacked capacity in terms of the workforce to cope with such peaks. Clinics and schools often served as camps for displaced people. Skills in emergency medicine and injuries were needed during the acute event. During acute shocks primary care providers needed skills to manage infectious disease outbreaks. Psychosocial assistance was also needed for people who had lost their families, livelihoods and homes.

If services were destroyed, then people relied on help from outside and other regions. Having more mobile health facilities could enable adaptation to climate events and maintain access for the community. Sometimes there was a need to deploy field hospitals.

### Issues with routine service delivery

Primary care providers needed the ability to respond to emergencies while also maintaining routine services for immunisations and chronic conditions such as HIV, diabetes and hypertension. Services for pregnant women and intrapartum care needed to be maintained. The availability of resources and an intact supply chain was important.

Implementing community-orientated primary care (COPC) was seen as appropriate to prepare for climate change. This included community engagement to inform communities and raise awareness of the issues. This could also lead to discussion on how to prepare and develop solutions and build community leadership. The COPC approach also emphasised intersectoral collaboration and the inclusion of local government, traditional leaders and universities in building community resilience. This approach would require a paradigm shift in the minds of healthcare workers and the need for training.

### Issues with climate resilience

Respondents mostly reflected on climate resilience and not environmental sustainability. Facility infrastructure had not been designed with climate hazards in mind and buildings were not able to withstand the impact of cyclones and floods. In other settings patients might have to wait outside in high temperatures without adequate shade, which also put them at risk.

Energy was an important issue, particularly for the cold chain and vaccines. In some remote and rural areas there was never mains electricity supply and solar might be installed, but even this could be broken. In general, it appeared that the focus was on alternative power supplies and not renewal or clean energy options. As temperatures rose, facilities installed more air conditioning units and used them for longer, which increased the need for energy and their carbon footprint.

Facilities experienced problems with procuring and repairing essential equipment such as batteries and ensuring sufficient stock for emergencies. Medication supplies and lack of resources quickly become an issue in disaster situations. High temperatures could also affect equipment, for example one respondent reported that his stethoscope had melted. Operating in full surgical protection in high temperatures was challenging. Mattress covers could also become very hot increasing the risks of bed sores.

Respondents recognised that the staff were as much at risk as the community members and in climate hazards staff also died. Climate events, such as cyclones and floods, could prevent both staff, patients and emergency services from reaching facilities. Extreme heat could also reduce access for rural patients who had to walk to facilities and were unwilling to expose themselves during the day.

Climate events and stressors could also impact on safe water supplies and sanitation at the facility as well as in the community, especially in rural and remote areas. Not all facilities had water storage or sufficient storage to last during interruption of supplies.

Respondents rarely reflected on issues of environmental sustainability, but one or two acknowledged the need for behaviour change regarding plastic and paper waste. Burning waste such as plastic was recognised as an environmental hazard that contributed to air pollution.

### Approaches to education

#### Pre-service education

Most respondents supported the idea of including education on climate change into undergraduate education. They believed that health professionals should be more aware of the issues when they enter practice, and such education might also raise awareness amongst their trainers. Education should, therefore, include the health and social effects of climate change as well as climate resilience and environmental sustainability of facilities and services.

#### Continuing professional development

Respondents reported that CPD could happen at different levels:

- At the local level many health facilities organised brief face-to-face CPD or journal club meetings.
- At a national level CPD was often provided by professional bodies. However, CPD may not be specific for primary care providers, particularly if there was not an active professional body. On-line training opportunities were preferred with short courses (1–2 days), webinars or journal articles. The COVID-19 pandemic had enabled the widespread use of web-based CPD, particularly webinars. In one country, obtaining a certificate from a short course was seen as an incentive. Online short courses were easily accessible and asynchronous material could be accessed after hours or at weekends. Most primary care providers had smartphones but might not have laptops, which could limit the types of educational activities. For example, written assignments were difficult on a smartphone. However, such courses should support participants to apply their learning to their own practice and community context. In some settings, face-to-face activities were incentivised by per diems from donor organisations and opportunities to take time out of practice. This raised expectations among primary care providers of remuneration to attend CPD.
- At a regional level, there was value in networks such as PRIMAFAMED raising awareness and giving opportunities for people to share experiences, resources and solutions from different countries.

Respondents thought that CPD needed to be informed by more research and evidence coming from the African setting. Countries that required CPD had more buy-in from health professionals and could even potentially require professionals to attend CPD on climate change. All respondents agreed that climate change had not yet been addressed in their CPD programmes.

#### Key topics for continuing professional development

The effects of climate change were seen across the whole burden of disease and information could be integrated into the usual clinical CPD topics, such as infectious diseases (e.g. malaria, gastroenteritis) and non-communicable diseases (e.g. diabetes, hypertension). Injuries and emergency medicine, maternal care, child health and respiratory tract infections were also mentioned. More specific topics included:

- Understanding the pathways by which climate change impacts different diseases and conditions
- Thinking about the health and social effects of extreme heat
- Understanding how climate change impacts the population at risk through the social and environmental determinants of health. This was linked to a shift in mindset that embraced COPC.
- Disaster preparedness and management, including psychosocial counselling and mental health issues and the role of primary care
- How to make your practice or facility more climate resilient and environmentally sustainable. The latter could include a focus on prescribing, anaesthesia and medical waste disposal.
- How to engage and educate patients and communities on climate related health issues. One or two respondents thought that family physicians should have advocacy skills to engage policymakers.

#### Resources needed for continuing professional development

Many people highlighted the value of open access journal articles as a key resource for CPD. While including climate change in textbooks could also be valuable, these quickly become outdated and were less accessible. The use of multiple-choice questions linked to journal articles was not emphasised. Having expert speakers available to present at webinars could stimulate interest in the target audience. Making electronic CPD resources available could be helpful, such as video clips, podcasts or PowerPoint slides.

## Discussion

### A summary of key findings

The findings suggested that primary care providers had six broad learning needs as shown in Table 3. Many of the clinical effects of climate change could be considered within the usual CPD topics. CPD needed to be supported at the micro-, meso- and macro-levels of the health system. For example, through facility-based journal clubs, national CPD programmes or regional network meetings. Different educational approaches could be supported by a menu of resources such as journal articles with accompanying digital resource materials accessed via a smart phone.

**TABLE 3:** Key learning needs of African primary care providers.

Number	Learning needs
1	Awareness of the pathways that link climate change to important health and social effects and changes in the management of diseases and conditions
2	Management of diseases linked to exposure to extreme heat
3	Development of a community-orientated primary care approach that includes attention to climate change and environmental determinants of health
4	Disaster preparedness and management in primary care
5	How to make your facility and services more climate resilient and environmentally sustainable
6	How to engage and educate patients and communities on climate related health issues

### Discussion of key findings

Most of the available frameworks and guidelines speak to the integration of planetary health education into formal curricula, particularly at the undergraduate level.^12,13^ In the African context, primary care providers have had limited exposure to planetary health thinking in their undergraduate training and exposure at the post-graduate level is also unlikely. Continuing professional development must therefore assume little or no prior knowledge and skills. On the other hand, many primary care providers have experienced the health and social effects of climate change, even if they have not attributed such effects to climate change.

A recent scoping review of eco-nursing competencies outlined five broad areas^14^: advocacy, research, education, clinical practice and leadership. Our findings did not emphasise research, advocacy or education, but mainly focused on learning needs related to clinical practice and leadership. Even within these areas, our respondents emphasised different topics such as disaster and emergency preparedness, resilience of facilities and services and strengthening a COPC approach.

There was also a significant mismatch with the skills for planetary health promoted by the AMEE guideline.^10^ This guideline emphasises four key skills: epidemiology, promoting health lifestyles, supporting pathways to net zero healthcare and eco-ethical leadership. Our respondents did not emphasise mitigation for carbon neutrality but rather the need for resilience. The African contribution to the global carbon footprint is negligible,^15^ while vulnerability to the challenges of climate change was in the foreground of people’s experience. Most African primary care providers are not involved with research and have little electronic or big data.^16^ Contributing to epidemiology and the evidence-base was not therefore a felt need although respondents did recognise the need for more evidence to guide education.

The planetary health education framework outlines five core domains^17^: interconnection with nature, the Anthropocene and health, systems thinking and complexity, equity and social justice, movement building and systems change. These principles could provide a useful lens to develop practical educational resources in line with the expressed learning needs in this study. For example, in understanding our interconnection with nature and the effects of climate change we should recognise African indigenous and traditional knowledge. In exploring the Anthropocene and health, we should think about One Health and how human and animal health are intertwined.^18^ Pastoralism is still very strong in African communities, and climate change also increases confrontation with wildlife.^19^ A community-orientated approach requires complex systems thinking to address social and environmental determinants.^20^ Respondents identified various vulnerable groups and the need to build equity and social justice into emergency preparedness and access to health care.

### Strengths and limitations

The key informants were medical practitioners in the fields of family medicine, primary care and public health. Even these key informants sometimes struggled to reflect on their experiences and translate them into learning needs related to climate change. This thinking is new for everyone. The voices of nurses and clinical officers were not directly included in this study and their perspective may be different, although the practitioners interviewed are leaders of primary care teams in their various contexts.

We believe that sufficient saturation of themes was achieved to take the study to the next stage. Results may be broadly transferable to public sector primary care contexts in sub-Saharan Africa, although francophone countries were not included.

### Implications

This study is part of a sequential exploratory mixed methods study and in the next phase we want to survey members of the PRIMAFAMED network across the region on the topics and educational approaches identified here. Following this survey, we intend to develop educational resources that can address the learning needs through CPD activities.

## Conclusion

Primary care providers have learning needs related to developing a conceptual understanding of the health and social effects of climate change, management of climate-sensitive clinical conditions, implementation of COPC, disaster management and emergency preparedness, building climate resilient facilities and services, and engaging patients and communities on these same issues. Some of these needs can be addressed through the usual CPD, but resources need to be developed for more specific topics. Respondents suggested the use of journal articles, supported by digital resources, which could be accessed on smart phones and enable local CPD. The findings will be further validated and quantified in a survey of primary care providers.
